# Expression of DinJ-YafQ System of *Lactobacillus casei* Group Strains in Response to Food Processing Stresses

**DOI:** 10.3390/microorganisms7100438

**Published:** 2019-10-11

**Authors:** Alessia Levante, Claudia Folli, Barbara Montanini, Alberto Ferrari, Erasmo Neviani, Camilla Lazzi

**Affiliations:** 1 Department of Food and Drug, University of Parma, 43124 Parma, Italy; alessia.levante@unipr.it (A.L.); alberto.ferrari2@studenti.unipr.it (A.F.); erasmo.neviani@unipr.it (E.N.); 2 Department of Chemistry, Life Sciences and Environmental Sustainability, University of Parma, 43124 Parma, Italy; barbara.montanini@unipr.it

**Keywords:** DinJ-YafQ, toxin-antitoxin systems, *L. casei* group, food-processing stress, RT qPCR, gene expression

## Abstract

Toxin-antitoxin (TA) systems are widely distributed in bacterial genomes and are involved in the adaptive response of microorganisms to stress conditions. Few studies have addressed TA systems in *Lactobacillus* and their role in the adaptation to food environments and processes. In this work, for six strains belonging to *L. casei* group isolated from dairy products, the expression of DinJ-YafQ TA system was investigated after exposure to various food-related stresses (nutrient starvation, low pH, high salt concentration, oxidative stress, and high temperature), as well as to the presence of antibiotics. In particular, culturability and DinJ-YafQ expression were evaluated for all strains and conditions by plate counts and RT qPCR. Among all the food-related stress conditions, only thermal stress was capable to significantly affect culturability. Furthermore, exposure to ampicillin significantly decreased the culturability of two *L. rhamnosus* strains. The regulation of DinJ-YafQ TA system resulted strain-specific; however, high temperature was the most significant stress condition able to modulate DinJ-YafQ expression. The increasing knowledge about TA systems activity and regulation might offer new perspectives to understand the mechanisms that *L. casei* group strains exploit to adapt to different niches or production processes.

## 1. Introduction

Toxin-antitoxin (TA) are systems encoded by two neighbouring genes widely distributed in the genomes of bacteria and archea, involved in the management of adaptive response of microorganisms by regulating cell growth and death as a result of stress conditions. All the TA systems currently known involve a stable toxin, that causes bacterial growth inhibition or bacterial death by interfering with essential cellular processes, and a cognate antitoxin capable of repressing toxin activity. TA systems are assigned to six classes, numbered I-VI, according to the nature of the antitoxin and its mode of action [1]. Activation of TA systems might trigger bacteria into a persistence state, by slowing down replicative machinery or interfering with protein synthesis, thus diverting cellular resources towards survival [2]. The persistent state is widely studied as a bacterial response to the presence of antibiotics but it has been also associated to abiotic stress conditions [2]. Despite their wide dissemination [3,4], few studies have addressed TA systems in the genus *Lactobacillus*, which is extensively distributed in a variety of habitats (food, feed, plants, vertebrate gastrointestinal tract), and used for different biotechnological processes and industrial applications [5]. In particular, the species *L. casei*, *L. paracasei* and *L. rhamnosus*, commonly defined as *L. casei* group due to their phylogenetic relatedness, are associated with a variety of environments, such as raw and fermented dairy products, fermented vegetables and gut microbiota of vertebrates [5]. Their ubiquity in diverse environments is due to their metabolic flexibility [6], such as the aptitude to ferment different substrates [7,8], but also to their capability to cope with environmental stress conditions commonly encountered in their niches, such as food environment [9,10] and human gastrointestinal tract. Interestingly, metagenome analysis of human gut microbiome has shown an enrichment of TA modules, suggesting a role of these genes in microbial colonization of human gut [11,12]. Few studies have described the presence of TA loci in strains belonging to the species *L. rhamnosus* and *L. paracasei*, isolated from food matrices or from human hosts. In particular, the type I TA locus identified in plasmid DNA of dairy *L. rhamnosus* isolates, is characterized by a mRNA encoding for the toxin peptide (Lpt) and a non-coding RNA acting as antitoxin convergently transcribed [13]. Various type II TA systems have been identified in dairy and human *L. casei* group isolates [14,15,16]. All the identified type II toxins (YafQ, YoeB, RelE and MazF) act as mRNA interferases, resulting in the inhibition of protein synthesis [17]. Overall, expression of the identified toxins in *E. coli* inhibits cell growth to various degrees but, in few cases, conserved toxins had lost their function [14,15]. The presence of TA systems in *L. casei* group might promote bacterial survival in the presence of stress conditions that are often encountered in food production and fermentation processes. This phenomenon has been observed for *L. casei* group in response to raw-milk cheese manufacturing process characterized by technological parameters inducing a selective pressure (low pH, high salt concentration, oxidative stress, high temperature) [18]. In this case, the initial bacterial load is low and increases during the ripening despite the unfavourable growth environment.

Ferrari et al. [14] described DinJ-YafQ systems found in dairy isolates of *L. rhamnosus* and *L. paracasei*, revealing the existence of homologous YafQ proteins characterised by different toxic activity. YafQ toxin and its cognate antitoxin DinJ are encoded within an operon, with the antitoxin gene (*din*J) located upstream of the toxin gene (*yaf*Q) [14]. In the present work, some of *L. paracasei* and *L. rhamnosus* strains harbouring the *din*J-*yaf*Q operon identified previously [14], have been exposed to various food processing related stresses as well as to a common stress, such as the presence of antibiotics, to assess which conditions can influence the expression of DinJ-YafQ transcript.

## 2. Materials and Methods

### 2.1. Bacterial Strains and Culture Conditions

Three strains of *L. paracasei* (2333, 4186, 4366) and three strains of *L. rhamnosus* (1019, 1473, 2360), isolated from dairy matrices, and belonging to the collection of the Laboratory of Food Microbiology of the Department of Food and Drug of the University of Parma, were used for this study (Table 1). *L. paracasei* ATCC 334 was purchased from ATCC and used as reference strain for housekeeping gene selection. Bacterial strains were maintained as frozen stocks (−80 °C) in Man Rogosa Sharpe (MRS) medium (Oxoid Limited, Basingstoke, UK) supplemented with glycerol 15% (*w*/*v*). The cultures were propagated three times with a 2% (*v*/*v*) inoculum in MRS and incubated in anaerobiosis (AnaeroGen, Oxoid, Basingstoke, UK) at 37 °C for 15 h.

### 2.2. Sequence Analysis

The nucleotide sequences of *L. rhamnosus* 2360 and *L. paracasei* 4366 were amplified by PCR under standard conditions by using *Lactobacillus* total DNA as template, the primers reported in Table 2 and GoTaq DNA polymerase (Promega Corporation, Madison, WI, USA). Nucleotide sequences of all the other strains derived from an ongoing genome sequence project. Nucleotide or amino acid multiple sequence alignments were performed by using MEGA7 software [19] and ClustalW algorithm [20]. GeneDoc, version 2.7 [21] was used for multiple sequences alignment representations.

### 2.3. Culture Conditions for Gene Expression Studies

Bacterial strains were grown overnight in batch culture as described above, and then inoculated at 2% (*v*/*v*) in 160 mL of fresh MRS broth preheated at 37 °C. Cells were grown until the beginning of the stationary phase (OD600 = 0.8), and subsequently divided into 10 mL aliquots. Cells were harvested by centrifugation for 10 min at 8200 g at 37 °C, MRS medium was removed and replaced with 10 mL of the same fresh medium for the control sample, or either with one of the media conveying the stress conditions reported in Table 3. The concentrations used for ampicillin (0.1 mg/mL) and for kanamycin (0.05 mg/mL) were lower than the MIC values calculated for all the strains for both antibiotics (ranging from 0.39 to 1.56 mg/mL for ampicillin and from 1.56 to 6.25 mg/mL for kanamycin). Cultures were incubated at 37 °C, or in a water bath at 55 °C, corresponding to the temperature reached during cheese-making process. For all the tested conditions, incubation proceeded for 30 or 120 min. To evaluate DinJ-YafQ expression after thermal stress relief, samples were incubated at 55 °C for 30 min and subsequently transferred to 37 °C for 90 min. Each experiment was performed in duplicate using different culture propagations, 1 mL of cell culture was used for bacterial culturability assays, performing decimal dilutions in Ringers solution (Oxoid, Basingstoke, UK), followed by plating in duplicate on MRS and incubation at 37 °C for 48 h. Bacterial culturability is reported as Log CFU/mL from the results of the mean values from the two biological replicates. The remaining culture was centrifuged for 5 min at 18,500× *g* at 4 °C, the supernatant was removed and the bacterial pellet stored at −80 °C prior to RNA extraction. *L. paracasei* ATCC 334, used as type strain for reference gene selection, was exposed to stress conditions for 30 min.

### 2.4. RNA Extraction and cDNA Synthesis

Bacterial pellets were re-suspended in RLT buffer (QIAGEN, Hilden, Germany), transferred to screw-cap tubes containing 250 mg of 0.1 mm zirconia/silica beads (BioSpec Products, Bartlesville, OK, USA) and disrupted by bead beating for 1 min for four times, each followed by 1 min on ice, using a Mini-Beadbeater-8 instrument (BioSpec Products, Bartlesville, OK, USA). RNA was then extracted with RNeasy Protect Bacteria Mini Kit (QIAGEN, Hilden, Germany), following manufacturer’s instructions. Integrity of the RNA sample was verified on 1.5% denaturing agarose gel. Total RNA was treated with Turbo DNA-free kit (Thermo Fisher Scientific, Waltham, MA, USA), RNA concentration and purity were measured using NanoDrop 2000 (Thermo Fisher Scientific, Waltham, MA, USA), and 1 µg of RNA was reverse transcribed with SuperScript IV Reverse Transcriptase (Thermo Fisher Scientific, Waltham, MA, USA) using random hexamers, according to the manufacturer’s protocol.

### 2.5. Reference Gene Selection

16S rRNA was used as a reference gene in real time quantitative PCR (RT qPCR) experiments aimed to analyze DinJ-YafQ expression for all the strains, with the exception of *L. paracasei* 4366. For this strain, due to the extensive degradation of 16S rRNA observed after incubation at 55 °C, an alternative set of reference genes was tested. On the basis of data reported in the literature [23,24], three genes, coding for glyceraldehyde-3-phosphate dehydrogenase (*gapdh-1*), gyrase B (*gyrB*) and recombinase A (*recA*) were considered in comparison with 16S rRNA. *gapdh*-1, *gyr*B and *rec*A were tested in *L paracasei* ATCC 334 exposed to stress conditions by using primers designed on the *L. casei* group sequences available in NCBI databank. A multiple alignment was performed using MEGA7 [19], and, where necessary, degenerate primers were designed to account for sequence heterogeneity over the three species. Standard curves, primer efficiency (E%) and correlation coefficients (*R^2^*) for all primer pairs were determined as described in the next section and are reported in Table 4. Selection of the reference gene was performed using the gene stability score (M), a metric designed to identify the most stable endogenous control gene among a set of samples [25].

### 2.6. Relative Quantification of DinJ-YafQ Expression

RT qPCR experiments were carried out on biological duplicates (including two technical replicates for each sample) by using the QuantStudio® 3 PCR system (Thermo Fisher Scientific, Waltham, MA, USA) and the PowerUp™ SYBR® Green Master Mix (Thermo Fisher Scientific, Waltham, MA, USA). The 20 µL PCR reaction included 0.5 µL of cDNA, 1 µM of forward and reverse primers and 10 µL of Master Mix solution. The reactions were incubated at 95 °C for 10 min, followed by 40 cycles of 95 °C for 15 s and 60 °C for 1 min. *C*t data were determined using default threshold settings, and the mean *C*t were determined from the two PCR technical replicates. The 2^−ΔΔ*C*t^ method was used to determine the relative gene expression [26] using 16S rRNA as a reference gene, except for *L. paracasei* 4366, for which all the experiments were repeated using *gyr*B as a reference. The reported relative gene expression values are the average of values obtained from the two biological duplicates. The real-time PCR amplification efficiencies (E%) were assessed for each primer pair on decimal dilutions of total DNA extracted from the bacterial strains, over 6 orders of magnitude, and calculated according to the equation: E% = (10^(−1/slope)^ − 1) × 100 (Table 4).

### 2.7. Statistical Analysis

DinJ-YafQ expression data, were statistically analyzed using SPSS Statistics 21.0 software (SPSS Inc., Chicago, IL, USA). Statistical differences on measured culturability for each strain were evaluated among all tested conditions using one way ANOVA, followed by two-sided Dunnett-t-test using the control condition as a reference. Statistical significance between gene expression data obtained for strain *L. paracasei* 4366 using either 16S rRNA or *gyr*B as a reference gene was calculated using Student’s T-test. DinJ-YafQ expression data for each strain were compared among all tested conditions using one way ANOVA. Two-sided Dunnett-t-test was adopted for post-hoc analysis, choosing the control condition as a reference. Data were considered significantly different when *p* < 0.05.

## 3. Results

### 3.1. Sequence Analysis

**Figure 1:**
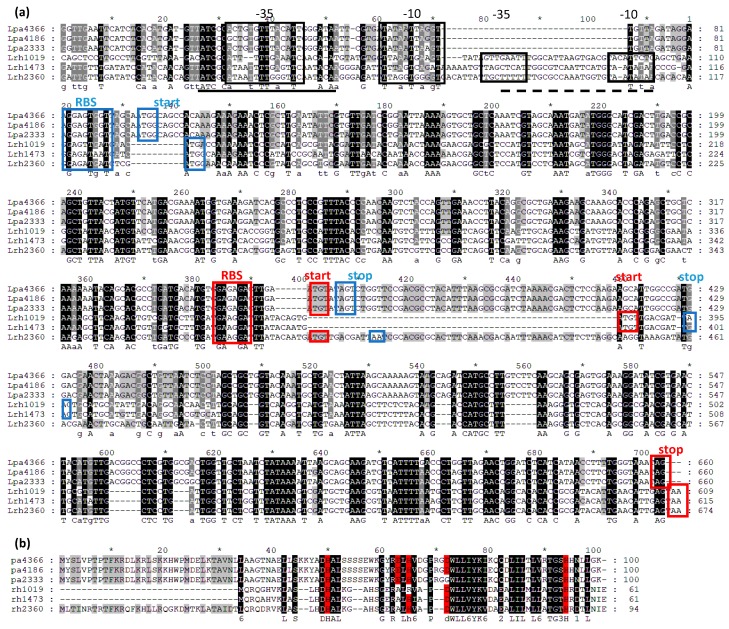
(**a**) Multiple alignment of nucleotide sequences including *din*J-*yaf*Q operons and their promoter regions from all the *Lactobacillus* strains used in this work. Predicted promoter regions are boxed in black and palindromic sequence are indicated by black line for *L. paracasei* sequences and by dashed black line for *L. rhamnosus* sequences. The ribosome binding site (RBS), the start codon (start) and the stop codon (stop) are boxed in blue and red for *din*J and *yaf*Q sequences, respectively. (**b**) Multiple alignment of amino acid YafQ sequences from all the *Lactobacillus* strains used in this work. Catalytic residues, as identified in *E. coli*, are highlight in red.

Nucleotide sequences comprising *din*J-*yaf*Q operon, identified in all the bacterial strains used in this work, were aligned to evaluate the organization of the promoter regions (Figure 1a). Only one promoter was identified in *L. paracasei* strains, while two possible promoters were predicted upstream *din*J-*yaf*Q operons of *L. rhamnosus* strains. In the promoter regions are also located palindromic sequences characteristically present upstream dinJ-yafQ operons [27] that might bind *Lactobacillus* DinJ or DinJ-YafQ complex. The two sequences encoding for DinJ and YafQ proteins are partially overlapped and preceded by a ribosomal binding site. The comparison among YafQ amino acid sequences, shown in Figure 1b, highlights the residues crucial for the enzymatic activity of the YafQ toxin, as they have been identified in *E. coli* protein [28].

In a previous work [14], the toxic activity of YafQ proteins from *L. rhamnosus* 1473 and 2360 and from *L. paracasei* 2333 and 4366 was verified in *E. coli*. YafQ from *L. rhamnosus* 2360 and from *L. paracasei* 4366 were able to inhibit cell growth and to influence RNA metabolism, while YafQ from *L. rhamnosus* 1473 and from *L. paracasei* 2333 did not show toxic activity.

As shown in Figure 1b, in both YafQ from *L. paracasei* 4366 and 4186 all the catalytic residues are conserved, while in the strain 2333, the replacement of a conserved aspartate residue with a glycine is probably responsible for the lack of its activity. YafQ from *L. rhamnosus* 1473 and 1019 are both characterized by a N-terminal truncated form, that probably prevents the toxic activity, as demonstrated for YafQ isolated from the strain 1473.

### 3.2. Bacterial Culturability in Response to Food Processing Stresses and to the Exposure to Antibiotics

**Figure 2:**
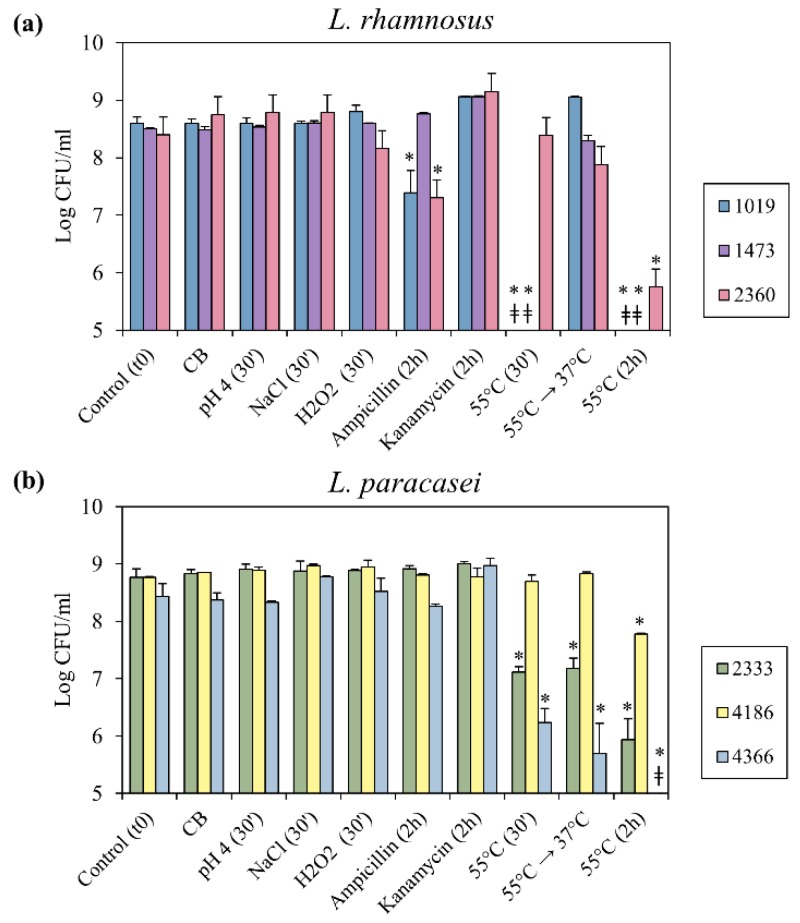
Bacterial culturability after exposure to stress conditions. Plate counts of (**a**) *L. rhamnosus* strains, (**b**) *L. paracasei* strains exposed to the indicated stress conditions. ‡, bacterial loads below the limit of detection (set at 5 Log CFU/mL); lower dilution (until 10^−2^) were assayed for these samples, but no colonies were observed after 48 h of incubation. Error bars represent the standard error of the mean (SEM, *n* = 2). * significant difference, *p* < 0.05.

Taking into account that all *L. paracasei* and *L. rhamnosus* strains used in this work were isolated from dairy products (milk or cheese, Table 1), bacterial culturability was evaluated after exposure to stress conditions related to cheese-production process, such as nutrient starvation, low pH, high salt concentration, oxidative stress and high temperature. In particular, nutrient starvation condition is simulated by Cheese Broth (CB) media, characterized by a composition that mimics ripened cheese environment [22]. Furthermore, by considering that *L. casei* group bacteria could be able of respiratory growth, the effect of oxidative stress condition was verified in the presence of H_2_O_2_. Finally, the effect of incubation in the presence of antibiotics representative of two different classes (ampicillin and kanamycin) on cell culturability was also verified as a common stress condition for the bacterial cell. Cultures were grown until late exponential growth phase prior to application of the selected stress condition. For each experimental setting, plate counts on MRS agar were performed to determine the bacterial culturability after exposure to potentially stressful conditions. As shown in Figure 2, exposure of the bacterial cultures to the food-related stress conditions led to a negligible variation of bacterial culturability compared to the control condition, with the exception of thermal stress conditions. In the case of *L. rhamnosus*, the exposure to 55 °C for 30 min led strains 1019 and 1473 to a loss of culturability greater than 4 Log CFU/mL, while the strain 2360 did not show a relevant variation (Figure 2a). When the incubation time at 55 °C was prolonged for two hours, strain 2360 culturability decreased of 2.6 Log CFU/mL (Figure 2a). For *L. paracasei*, after 30 min at 55 °C, culturability of the strain 4186 did not change, while a reduction of more than 1.6 Log CFU/mL was observed for 2333 and 4366 strains (Figure 2b). The prolonged incubation at 55 °C caused a significant reduction of bacterial load for all *L. paracasei* strains (Figure 2b). Finally, when samples exposed to 55 °C for 30 min were transferred to 37 °C for 90 min, only *L. rhamnosus* 1019 and 1473 recovered culturability values comparable with those of the control (Figure 2a). The exposure to antibiotics showed different outcomes on bacterial culturability. The presence of ampicillin significantly decreased culturability of *L. rhamnosus* 1019 and 2360 (Figure 2a), while exposure to kanamycin did not affect any strain.

### 3.3. Selection of a Reference Gene for Relative Expression Studies

**Figure 3:**
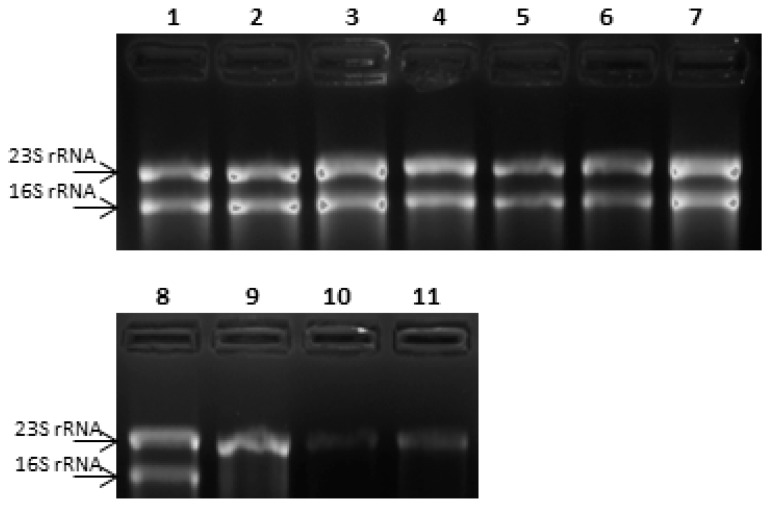
16S rRNA degradation after thermal stress conditions. Denaturing gel electrophoresis of total RNA extracted from *L. paracasei* 4366. 1, control; 2, nutritional stress; 3, acidic stress; 4, osmotic stress; 5, oxidative stress; 6, ampicillin; 7, kanamycin; 8, control; 9, thermal stress (30 min); 10, thermal stress relief; 11, thermal stress (2 h). Black arrows indicate the 23S rRNA and16S rRNA.

As evaluated on denaturing agarose gel electrophoresis, RNAs, extracted from bacterial samples exposed to different stress conditions, showed good levels of RNA integrity, except for RNA extracted from *L. paracasei* 4366 subjected to thermal stress conditions. In this case, total RNA showed an extensive degradation of the 16S rRNA (Figure 3), confirmed by a reduction of the *C*t values measured by RT qPCR using TBA primers (Table 4). Therefore, 16S rRNA could not represent a suitable reference gene for *L. paracasei* 4366. To evaluate alternative reference genes for relative expression analysis, a preliminary study was performed in *L. paracasei* ATCC 334, the type strain for this species. This strain was exposed to the same stress conditions previously described, and the expression levels of 16S rRNA, *gyrB*, *recA* and *gapdh*-1 genes were measured by RT-qPCR. *G*ene-stability score (M), was calculated for each gene in comparison with 16S rRNA (Table 5). *gyr*B showed the lowest M score (0.47), resulting the best candidate reference gene. Furthermore, the use of multiple control genes for calculation did not lead to a decrease of M value (data not shown).

**Figure 4:**
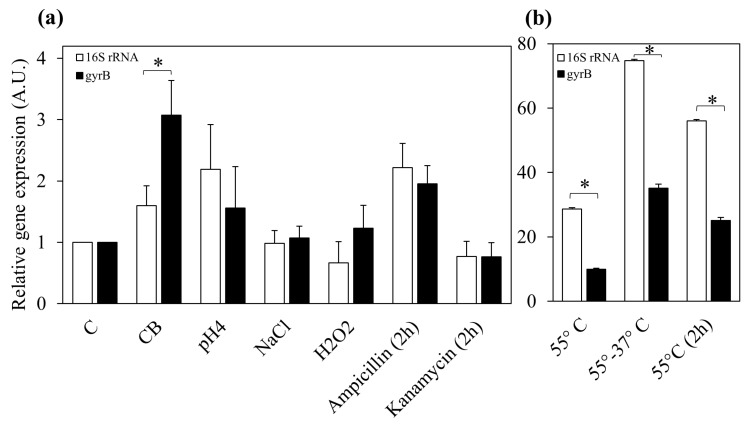
Relative expression of DinJ-YafQ transcript in *L. paracasei* 4366 measured using 16S rRNA (white) or *gyr*B (black) as control genes. (**a**) Relative expression in response to nutritional, acidic, osmotic, oxidative stress conditions and in response to exposure to antibiotics, (**b**) Relative expression in response to thermal stress. DinJ-YafQ relative expression is reported as fold changes vs. control (C) condition. Error bars represent the SEM (*n* = 2). * significant difference, *p* < 0.05.

To further validate the use of *gyr*B as reference gene in *L. paracasei* 4366, the relative expression of DinJ-YafQ transcript was evaluated after exposure of bacterial cultures to the same stress conditions, using either 16S rRNA or *gyr*B as a reference gene (Figure 4). The measured gene expression ratios did not vary significantly for most of the analyzed samples, indicating the integrity of 16S rRNA in these conditions. Under nutrient starvation condition (Figure 4a), DinJ-YafQ upregulation was higher when *gyr*B was used as a reference (*p* < 0.05), suggesting a variation in the expression of *gyr*B in this condition. When thermal stress conditions were taken into account (Figure 4b), normalization against *gyr*B caused the halving of the values, in comparison with 16S rRNA. In particular, DinJ-YafQ relative expression measured after 55 °C for 30 min resulted 9.9 ± 0.3 instead of 28.7 ± 0.3, and after two hours at 55 °C the relative expression values were 25.1 ± 0.7 instead of 56.1 ± 0.3. Finally, thermal stress relief condition showed a relative expression of 74.8 ± 0.4 using 16S rRNA as a reference, and 35.2 ± 1.2 using *gyr*B as housekeeper. Overall, using *gyr*B as a reference gene, a decreased relative expression was measured after exposure to heat stress conditions, further confirming that degradation of 16S rRNA caused the inflated values obtained using this gene as a reference.

### 3.4. Effect of Food-Related Stress Conditions and Exposure to Antibiotic on DinJ-YafQ Expression

**Figure 5:**
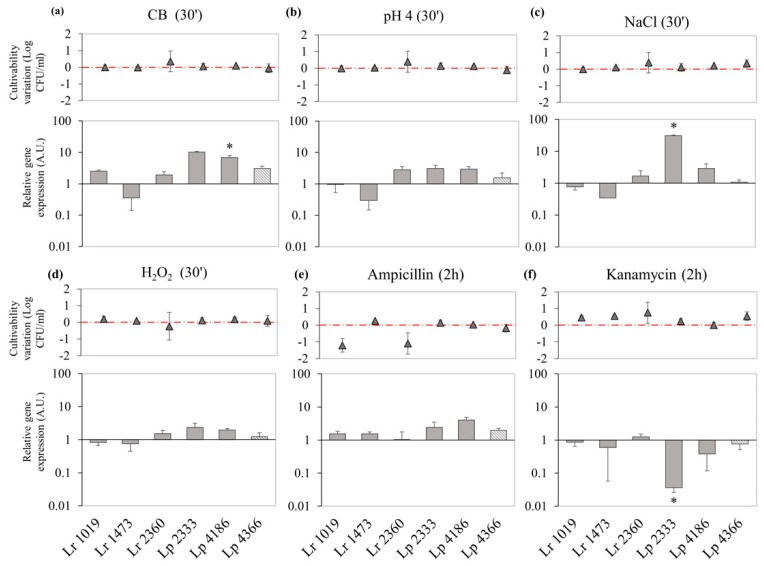
Relative expression of DinJ-YafQ transcript in *L. paracasei* (Lp) and *L. rhamnosus* (Lr) strains in response to food-related stress conditions, and exposure to antibiotics. The upper panels describe, for each strain, the culturability variation reported as the difference between the value measured under the stress condition and that measured in the control condition (grey triangles). The bar graph below reports relative gene expression calculated using 16S rRNA as a reference gene (gray bars), except for *L. paracasei* 4366 samples that were normalized using *gyrB* as a reference gene (striped gray bars). For all the strains gene expression was normalized against the control condition, that is not reported. (**a**) nutritional stress, (**b**) acidic stress, (**c**) osmotic stress, (**d**) oxidative stress, (**e**) exposure to ampicillin, (**f**) exposure to kanamycin. Error bars represent the SEM (*n* = 2). * significant difference, *p* < 0.05.

Bacterial cultures exposed to different stress conditions were analyzed for DinJ-YafQ expression. Nutritional stress led to a general overexpression of DinJ-YafQ system (Figure 5a) with the exception of strain 1473. In particular, *L. paracasei* 2333 and 4186 showed the greatest effect, with an upregulation of 10.3 ± 0.4 and 6.8 ± 1.2, respectively. Exposure to pH 4 for 30 min (Figure 5b) resulted in a slight downregulation of *din*J-*yaf*Q operon in *L. rhamnosus* 1473, while expression was slightly upregulated in *L. rhamnosus* 2360 and in all *L. paracasei* strains. Interestingly, exposure to 1.5% (*w*/*v*) NaCl led to upregulation of DinJ-YafQ expression in *L. paracasei* 2333 of 30.9 ± 1.2 folds. For the other strains a slight upregulation or downregulation was observed (Figure 5c). Oxidative stress (Figure 5d) led to no significant changes in relative expression of DinJ-YafQ. In all these conditions negligible differences were observed regarding culturability, as shown in the upper panels of Figure 4a–d and as previously described (Figure 2).

In the presence of the antibiotic ampicillin, *L. rhamnosus* and *L. paracasei* species showed no significant variations of DinJ-YafQ expression (Figure 5e, lower panel). Conversely, kanamycin led to a downregulated trend of DinJ-YafQ expression in almost all strains with the lowest expression ratio (0.03 ± 0.5) detected in *L. paracasei* 2333 (Figure 5f, lower panel). On the other hand, bacterial culturability was not affected (Figure 5f, upper panel).

Overall, we observed that the expression of DinJ-YafQ appears to be strain-specific under the analyzed stress conditions.

### 3.5. Effect of Thermal Stress on Bacterial Culturability and DinJ-YafQ Expression

Exposure to high temperature appeared to be the most significant stress condition able to regulate DinJ-YafQ expression.

**Figure 6:**
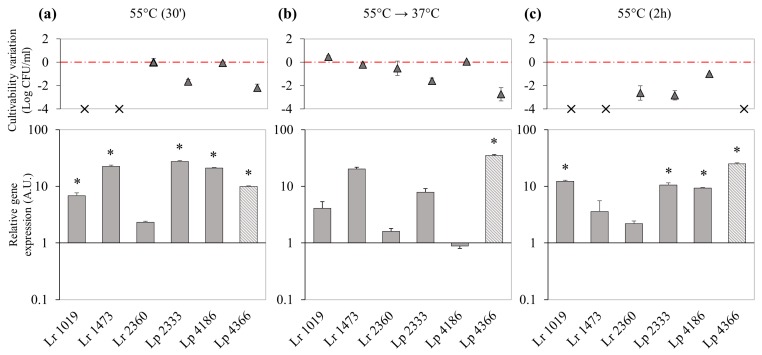
Relative expression of DinJ-YafQ transcript in *L. paracasei* (Lp) and *L. rhamnosus* (Lr) strains in response to thermal stress. The upper panels describe, for each strain, the culturability variation reported as the difference between the value measured under the stress condition and that measured in the control condition (gray triangles). The bar graphs below report relative gene expression calculated using 16S rRNA as a reference gene (gray bars), except for *L. paracasei* 4366 samples that were normalized using *gyrB* as a reference gene (striped gray bars). For all the strains gene expression was normalized against the control condition, that is not reported. (**a**) incubation at 55 °C for 30 min, (**b**) incubation at 55 °C for 30 min followed by incubation at 37 °C for 90 min, (**c**) incubation at 55 °C for 2 h. X symbols indicate a culturability below the chosen detection limit (5 Log CFU/mL); lower dilutions (until 10^−2^) were assayed for these samples but no colonies were observed after 48 h of incubation. Error bars represent the SEM (*n* = 2). * significant difference, *p* < 0.05.

When cell cultures were exposed to 55 °C for 30 min, culturability was impaired for *L. rhamnosus* 1019 and 1473, falling below the chosen detection limit (5 Log CFU/mL), and for *L. paracasei* 2333 and 4366, which showed a decrease of 1.6 ± 0.2 and 2.2 ± 0.3 Log CFU/mL, respectively (Figure 6a, upper panels). In this condition DinJ-YafQ expression appeared to be significantly upregulated in almost all the strains and *L. paracasei* 2333 showed the highest expression ratio (Figure 6a, lower panel).

When the cultures were moved to their optimal growth temperature, 37 °C for 90 min, *L. rhamnosus* 1019 and 1473 recovered their culturability, with values comparable with those of the controls, while, for the other strains, the culturability remained unchanged (Figure 6b, upper panel). In response to heat stress relief, expression of DinJ-YafQ module decreased in comparison with data obtained after incubation at 55 °C for 30 min with the only exception of *L. paracasei* 4366 in which the expression of DinJ-YafQ increased reaching an expression ratio of 35.2 ± 1.2 (Figure 6b, lower panel).

A prolonged exposure to heat stress (55 °C for 2 h), led to a decrease in culturability of two Log CFU/mL for *L. rhamnosus* 2360 and *L. paracasei* 2333, and of one Log CFU/mL for *L. paracasei* 4186, while for the other strains culturability decreased more than four Log CFU/mL (Figure 6c, upper panel). Comparing short and prolonged incubation at 55 °C, the expression in *L. rhamnosus* 2360 did not vary substantially, whereas for the other strains the expression slightly increased (strains 1019 and 4366) or decreased (strains 1473, 2333 and 4186), in a species independent manner.

## 4. Discussion

Bacterial TA systems could be activated upon exposure to a wide range of environmental stresses interfering with various cellular processes and inhibiting protein synthesis [1,17,29]. Under these conditions, bacterial metabolic activity could be arrested or slowed down permitting bacteria to survive in a hostile environment. Despite several studies reported in the literature investigate TA in pathogenic bacteria, only limited data are available on distribution and activity of TA systems in *Lactobacillus* genus and are focused on *L. casei* group [13,14,15,16]. The three species belonging to this taxonomic group, *L. casei*, *L. paracasei* and *L. rhamnosus*, are extensively used in food processes and are important components of the human gut microbiota. The presence of TA systems in these bacterial species could then contribute to bacterial fitness and resilience that are essential to survive in these environments.

*L. casei* species have been isolated in various niches and are characterized by genomic diversity and plasticity that represent the drive forces for a “nomadic” lifestyle [5]. In particular, the strains used in this work were isolated from various steps of a cheese-making process that uses raw milk and undefined starter culture for the production of a long ripened hard cheese [18]. During cheese manufacturing process a natural whey starter is added to raw milk. Following the curdling step, the curd is cooked at 53–56 °C for 40–70 min, exposing bacteria to a thermal stress. The product is then submerged in saturated brine that determines a final NaCl concentration in cheese of 1.5% (*w*/*v*). *L. casei* group strains are present at low concentration (1–2 Log CFU/mL) in raw milk and increase in number, up to four orders of magnitude, during the ripening. This phenomenon might be attributed to various strategies of adaptation [30], among which the activation of TA systems. Recently, Folli et al. [13] have observed that the cultivation of *L. rhamnosus* in a cheese-mimicking media led to the overexpression of the type I TA toxin Lpt, and its transcript could be retrieved in ripened cheese. Furthermore, Ferrari et al. [14] have characterized the distribution and activity of the type II TA system DinJ-YafQ in *L. casei* group strains. In particular, DinJ-YafQ TA system was identified in all the six strains used in this work and YafQ activity was investigated in four of them.

To evaluate the possible role played by DinJ-YafQ TA system on survivability in response to stress conditions, in this work we evaluated the relative expression of DinJ-YafQ transcript in bacterial strains exposed to food-related stresses (nutrient starvation, low pH, high salt concentration, oxidative stress, and high temperature), as well as to a common stress for the bacterial cells, such as the presence of antibiotics.

Overall, expression of DinJ-YafQ resulted to be not species-correlated but rather strain-specific. This inter-strain variability may be due to the high level of genomic heterogeneity among *L. casei* group [6,31]. Considering nutritional, acidic, osmotic and oxidative stresses, a negligible variation of culturability was observed for all the analyzed strains. In the same conditions, DinJ-YafQ transcript was upregulated at different levels for all the strains, with the exception of *L. rhamnosus* 1473 and 1019. By using cheese-mimicking conditions (CB media), characterised by absence of sugar and limited nutrient availability, almost all the strains showed a general increase of DinJ-YafQ expression as previously observed for Lpt toxin [13], suggesting that more than one TA system might be regulated as a response to nutrient starvation.

Notably, 1.5% (*w*/*v*) NaCl, mimicking the salt concentration in brined cheese, led to a significant increase of DinJ-YafQ expression for strain *L. paracasei* 2333, which harbors a non-toxic YafQ on the basis of toxicity assays performed in *E. coli* [14]. The regulation of the expression of this operon together with the high conservation of this toxin variant, which is present in about 20% of the *L. paracasei* sequences deposited in public databases, supports the hypothesis that this enzyme might have acquired a function other than mRNA interferase. Furthermore, DinJ antitoxin might regulate the transcription of other genes, involved in stress response, as suggested in [32].

Exposure to high temperature represents the stress condition that mainly influences culturability and *Lactobacillus* DinJ-YafQ expression level. *L. paracasei* were quite tolerant to thermal stress, while among *L. rhamnosus* the culturability of strains 1473 and 1019 decreased below the chosen detection limit already after 30 min of incubation at 55 °C. Anyway, the growth of these strains was restored when the thermal stress was relieved. Considering that both these strains harbor a truncated form of YafQ toxin [14], the ability to survive might be ascribed to other adaptive mechanisms, among which the activation of other TA systems. Regarding strain *L. paracasei* 4366, that harbors an active YafQ toxin, exposure to 55 °C for 30 min led to a decrease in culturability, accompanied by a significant upregulation of DinJ-YafQ. After relief of the thermal stress, the culturability did not recover, and the TA module was further upregulated. This phenomenon could be referred to the occurrence of transcriptional autoregulation observed in type II TA modules, that leads to an accumulation of YafQ toxin, while the antitoxin can be degraded by specific proteases, such as Lon-ClpP [1,33]. The endoribonuclease activity of YafQ might be linked to the extensive degradation of 16S rRNA observed in total RNA extracted from *L. paracasei* 4366 strain exposed to 55 °C and also to the lack of restoration of bacterial culturability, when thermal stress is relieved. To validate this hypothesis, future experiments will be directed to assess in vitro the ribonuclease activity of YafQ from *Lactobacillus* 4366. Under thermal stress condition, *L. rhamnosus* 2360, encoding an active variant of YafQ toxin, showed only a slight upregulation of the TA module, but no effect on culturability or extensive degradation of total RNA have been observed. These results suggest that YafQ toxin might be stored in the TA complex, thus not affecting cell physiology.

The exposure to antibiotics represents a typical stress condition that can trigger the entrance of bacterial cell in a non-dividing and metabolically inactive state by activation of TA systems, leading to the formation of persister cells that can evade antibiotic killing. A possible role in this mechanism has been previously proposed also for DinJ-YafQ system [32]. *L. paracasei* strains used in our experiments showed to be not susceptible to the tested concentrations of ampicillin and kanamycin but opposite DinJ-YafQ expression trends have been observed for all the strains when exposed to either one antibiotic. Concerning *L. rhamnosus*, a significant decrease in culturability was observed only in the case of 1019 and 2360 strains exposed to ampicillin, while a negligible variation in DinJ-YafQ expression levels was measured for all strains in the presence of ampicillin and kanamycin. 

Currently, only limited data are available on the expression regulation of TA modules in response to stress conditions. In this work, we studied how the expression of DinJ-YafQ systems identified in *Lactobacillus* strains isolated from the dairy niche is influenced by abiotic stressors. The selected stress conditions have been designed to simulate the environmental pressures that might shape the composition of microbial community during fermentation processes. Overall, our data suggest that, among the analyzed conditions, the expression of DinJ-YafQ is primarily regulated by exposure to thermal stress. To date, different mechanisms have been associated to thermal stress tolerance in *Lactobacillus* genus, and the increasing knowledge about TA systems regulation might offer new perspectives on these complex phenomena.

Since *L. casei* group bacteria are widely used in industrial applications and are important components of human gut microbiota, the comprehension of the regulation of TA modules expression could be of interest to understand the mechanisms that bacteria exploit to adapt to different niches or production processes.

## Figures and Tables

**Table 1:** Bacterial strains used in this study.

Strain	Species	Source	Ripening Time (Months)
ATCC 334	*L. paracasei*	Cheese	unknown
2333	*L. paracasei*	Cheese	6
4186	*L. paracasei*	Cheese	4
4366	*L. paracasei*	Raw cow’s milk	0
1019	*L. rhamnosus*	Cheese	4
1473	*L. rhamnosus*	Cheese	20
2360	*L. rhamnosus*	Cheese	13

**Table 2:** Primer pairs used in PCR amplification reactions.

Name	Sequence (5′–3′)	Strains	Reference
PLr2360 FW	CGGACAATTTTATATCGACCG	*L. rhamnosus* 2360	this work
dinj-yafQ_rh6 minus	TTACTCAATGTTCAATGTATCGCG		[14]
PLp4366 FW	ATACTATGTCGGTAAGGTCAG	*L. paracasei* 4366	this work
dinj-yafQ_ca4_pa3 minus	AAGGTTATGATGAGATCCGGTTC		[14]

**Table 3:** Culture conditions for DinJ-YafQ expression analyses.

Condition	Label	Media	Temperature (°C)	Time (min)
Control	C	MRS pH 6.4	37	0
Nutritional stress	CB	Cheese Broth (^1^)	37	30
Acidic stress	pH 4	MRS pH 4	37	30
Osmotic stress	NaCl	MRS pH 6.4, NaCl 1.5% (*w*/*v*)	37	30
Oxidative stress	H_2_O_2_	MRS pH 6.4, H_2_O_2_ 1 μM	37	30
Thermal stress	55 °C	MRS pH 6.4	55	30
	55 °C 2 h	MRS pH 6.4	55	120
Thermal stress relief	55–37 °C	MRS pH 6.4	55	30
			37	90
Antibiotic exposure	Amp	MRS pH 6.4, ampicillin 0.1 mg/mL	37	120
	Kan	MRS pH 6.4, kanamycin 0.05 mg/mL	37	120

^1^ [22].

**Table 4:** Primer pairs used for RT qPCR experiments.

Name	Sequence (5′–3′) ^1^	Target Gene	Species	E%	Slope	*R^2^*
GyrBFW	GCMCAGCCRCCGTTGTATCG	*gyr*B	*L. rhamnosus* *L. paracasei*	97.75	−3.38	0.999
GyrBRV	GYTGGCGTCCATTTCMCCAAG					
GapdH1FW	GTTGGTACCATGACCACCGT	*gapdh*-1	*L. rhamnosus* *L. paracasei*	103.65	−3.25	0.998
GapdH1RV	GTGCTGTGAGGAATCGTGTT					
RecAFW	GATGATGCACTTGGTGTTGG	*rec*A	*L. rhamnosus* *L. paracasei*	96.99	−3.40	0.999
RecARV	TCRGCATCAATATARGCGG					
TBAFW ^2^	CGGCAACGAGCGCAACCC	16S rRNA	*L. rhamnosus* *L. paracasei*	99.79	−3.33	0.999
TBARV ^2^	CCATTGTAGCACGTGTGTAGCC					
YafQ_lrFW	TGCAGCGTCAAGGTCATGTA	*din*J-*yaf*Q	*L. rhamnosus*	95.64	−3.43	0.998
YafQ_lrRV	CAATGTATCGCGGTGTGTGC					
YafQ_lpFW	GCCGATGGACGAACTAAAGA	*din*J-*yaf*Q	*L. paracasei*	98.76	−3.35	0.995
YafQ_lpRV	TATCCTTTCCACTCGCTGCT					

^1^ Degenerated bases, reported as indicated in IUPAC base coding, are underlined. ^2^ [23].

**Table 5:** *C*t range and gene-stability score (M) for the selected genes in *L. paracasei* ATCC 334.

Strain	Target	Min (*C*t)	Max (*C*t)	Range	M
ATCC 334	16S rRNA	7.62	9.54	1.92	–
	*gyr*B	17.18	19.79	2.61	0.47
	*gapdh*-1	13.10	17.54	4.44	1.36
	*rec*A	20.15	23.79	3.64	7.43
